# Genome Sequence of Escherichia coli Stbl4, a Versatile Genetic Tool for Heterologous Expression

**DOI:** 10.1128/MRA.00823-21

**Published:** 2021-10-07

**Authors:** Lina Assad, Karolis Matjošaitis, Harald Gross

**Affiliations:** a Department of Pharmaceutical Biology, Pharmaceutical Institute, University of Tübingen, Tübingen, Germany; b Thermo Fisher Scientific Baltics, Vilnius, Lithuania; c German Centre for Infection Research (DZIF), Partner Site Tübingen, Tübingen, Germany; University of Maryland School of Medicine

## Abstract

Escherichia coli Stbl4 is widely used as a laboratory strain for heterologous expression of large gene clusters. Since no genome sequence has been publicly available, we here report the draft sequence of Stbl4, including its F-plasmid. It should serve as a useful reference for researchers working with Stbl4.

## ANNOUNCEMENT

Historically, strain Stbl4 represents an advancement of Stbl2, which is derived from JM109/J5 (1). The latter strain was in turn constructed from strain DH1 (2, 3) via JM106 to JM108 (4). DH1 itself stems from the parent strain lineage strains MM294 (5), 1100 (6, 7), 1000 (7) and HfrCxW208 (8) from the original K-12 wild-type strain (8), which was isolated from the stool of a convalescent diphtheria patient in 1922 (9). The strain Stbl4 (shortcut for “stable 4”) is recommended for cloning of unstable DNA mediated by the introduction of repetitive DNA, retroviral sequences, or large inserts which otherwise would lead to undesired DNA rearrangements (10–12). For the latter reason, Stbl4 is also used as a host for the discovery of complex secondary metabolites (12).

During a heterologous expression study of a biosynthetic gene cluster (BGC) using Stbl4, we experienced unexpected cross talk between the inserted gene cluster and an existing BGC and needed detailed information about the genetic architecture of the host to investigate this phenomenon. Therefore, we describe the complete genome sequence of Stbl4.

ElectroMAX Stbl4 competent cells were purchased from Invitrogen and streaked onto Luria-Bertani (LB) agar plates containing tetracycline (12.5 μg/ml). After 16 h of incubation at 37°C, two single colonies were picked and inoculated into liquid LB at 37°C on a rotary shaker (200 rpm) until saturation. Genomic DNA extraction and PacBio library preparation were conducted as previously described (13). The 6-kb library was sequenced on a PacBio Sequel instrument using one single-molecule real-time (SMRT) cell. An aliquot of the same DNA preparation was used to create a genomic Nextera XT paired-end library for sequencing using an Illumina NovaSeq platform. The results of both sequencing platforms enabled a *de novo* hybrid assembly. The PacBio data were processed and filtered using the SMRT Link software suite, whereby subreads shorter than 50 bp were discarded. The remaining PacBio long reads were assembled using SMRT Link v7.0.1 and HGAP4 (14, 15). The initial quality assessment of the Illumina data was based on data passing the Illumina Chastity filter. Afterwards, reads containing a PhiX control signal were removed, and reads containing adapters were clipped. Further quality assessment was based on the remaining reads using the FASTQC tool v0.11.8 (16). Subsequently, the Illumina data were aligned to the abovementioned HGAP4 assembly genome using BBMap v36.77 (https://sourceforge.net/projects/bbmap/). Finally, assembly errors and the nucleotide disagreements between the Illumina reads and scaffold sequences were corrected using Pilon v1.21.1 (17). All software settings were kept at their defaults except for the HGAP4 genome size estimate parameter, which was set to 4.7 Mbp. Overall, the hybrid *de novo* assembly resulted in a nucleotide draft sequence consisting of two contigs, representing one chromosome and its F-plasmid (18, 19). The assembled contigs were annotated using the PGAP v5.2 pipeline (20, 21), while an automated secondary metabolism analysis was conducted employing antiSMASH v6.0.1 (22). The essential genome features are summarized in Table 1.

**TABLE 1:** Sequencing metrics for E. coli Stbl4

Statistic or characteristic	Data for E. coli Stbl4
PacBio sequencing	
No. of reads	540,276
Mean read length (bp)	4,673
No. of mapped reads	534,707
Avg coverage (×)	486
No. of contigs	6
*N*_50_ (bp)	4,404,505
No. of gaps	0
Illumina sequencing	
Read length (nt)^*a*^	2 × 150
No. of initial reads	14,301,826
Yield (Mbp)	2,049
No. of reads passing quality control	12,622,970 (88.3%)
Avg quality	35.68
Avg coverage (×)	431
Median insert size (bp)	354.00
*De novo* hybrid assembly	
Genome size (bp)	4,502,782
GC content (%)	50.7
No. of contigs	2
Contig 1 (chromosome) size (bp)	4,404,514
Contig 2 (F-plasmid) size (bp)	98,268
No. of genes (total)	4,353
No. of genes (coding)	4,060
No. of predicted biosynthetic gene clusters	2 (1 encoding thiopeptide, 1 encoding enterochelin)

a nt, nucleotides.

### Data availability.

The genome sequences of both contigs have been deposited in the NCBI GenBank database under accession numbers CP076043.1 and CP076044.1 for the chromosome and plasmid, respectively. The SRA accession numbers are SRX11660715 and SRR15358205 for the PacBio and Illumina reads, respectively.
